# Cost Effectiveness Analysis of an AI-Assisted Breast Cancer Screening Programme in Singapore: An Early Health Technology Assessment

**DOI:** 10.3390/cancers18050836

**Published:** 2026-03-04

**Authors:** Serene Si Ning Goh, Yuan Zheng Lim, Clarence Ong, Mikael Hartman, Yi Wang

**Affiliations:** 1 Saw Swee Hock School of Public Health, National University of Singapore and National University Health System, Singapore 117549, Singapore; clarence96@gmail.com (C.O.); ephbamh@nus.edu.sg (M.H.); ephwyi@nus.edu.sg (Y.W.); 2 Department of Surgery, Yong Loo Lin School of Medicine, National University of Singapore and National University Health System, Singapore 117597, Singapore; limyz@nus.edu.sg; 3 Department of Surgery, National University Hospital and National University Health System, Singapore 119228, Singapore

**Keywords:** breast screening, cost effectiveness analysis

## Abstract

**Background/Objectives**: This study assesses the cost-effectiveness of integrating artificial intelligence (AI) into breast cancer screening programs in Singapore. It evaluates AI as a standalone reader and as a companion reader alongside a consultant radiologist and compares these with double reading by two radiologists to determine economic viability and impact on healthcare resource use. **Methods**: A Markov model compared costs and outcomes of three strategies: double reading, a hybrid AI-assisted model (radiologist plus AI), and AI-only. These were applied to biennial mammography for 10,000 women aged 50–69 years in Singapore, with a 50-year horizon. Epidemiological and cost data were sourced from Asian and local studies and standardized to 2023 values, with a 3% annual discount. Outcomes were incremental cost-effectiveness ratios (ICERs) per quality-adjusted life-year (QALY). Deterministic and probabilistic sensitivity analyses assessed uncertainty. **Results**: Double reading cost USD 19.18 million with 218,460.4 QALYs. The AI-companion model cost USD 18.86 million with 218,476.3 QALYs, saving USD 316,090 and gaining 15.9 QALYs. The AI-only model cost USD 20.53 million with 218,532.4 QALYs, yielding 72.0 QALYs gained and an ICER of USD 18,743 per QALY. Specificity was the most influential parameter. At a willingness-to-pay threshold of USD 50,000 per QALY, AI-only screening had >75% probability of being most cost-effective. **Conclusions**: AI-assisted screening was cost-saving, while AI-only was cost-effective with greater health gains but higher costs and false positives. A phased, human-in-the-loop approach offers the most economically favourable strategy for AI integration.

## Simple Summary

Evidence on artificial intelligence in mammography has largely come from clinical trials, multi-reader evaluations, and national screening studies, showing improvements in cancer detection and reductions in radiologist workload without compromising safety. Despite these advances, no studies to date have assessed the cost-effectiveness of artificial intelligence integration into national screening programmes in an Asian setting. This study evaluates the cost-effectiveness of artificial intelligence-enhanced breast cancer screening under real-world conditions using a Markov model parameterized with Singapore-specific epidemiological, cost, and utility data. It shows that both artificial intelligence-assisted and artificial intelligence-standalone models can be cost-effective alternatives to conventional double reading. The artificial intelligence-assisted model delivers cost savings and health gains while retaining clinical oversight, while standalone artificial intelligence provides greater health gains but with higher costs and false positives. These findings provide context-sensitive evidence to guide policy, reimbursement, and integration of artificial intelligence into national screening programmes.

## 1. Introduction

Breast cancer is the most common cancer among women in Singapore and remains the leading cause of cancer-related mortality in females [1]. With rising incidence and improved survivorship, the burden extends beyond mortality to include long-term treatment costs and ongoing healthcare demands. To reduce late-stage presentations and improve outcomes, Singapore has implemented a biennial mammographic screening program under the Screen for Life initiative for women aged 50–69 years [2]. Mammography remains the only breast cancer screening modality with proven effectiveness, achieving over a 40% reduction in breast cancer mortality in high-income countries [3,4]. Despite this, sustaining an effective mammographic screening program poses several operational and diagnostic challenges. Mammogram interpretation is resource-intensive and relies heavily on a limited pool of trained radiologists. In Singapore, increasing screening volumes and the push for double-reading to improve accuracy have placed further strain on the radiology workforce. This pressure was particularly evident during the COVID-19 pandemic, when diagnostic imaging demands surged [5]. In low- and middle-resource settings, the reliance on human radiologists also raises questions of cost-effectiveness and scalability [6].

Interpretive variability and human fatigue contribute to missed cancers. Up to 16–31% of screen-detectable cancers may be missed by a single reader [7], and interval cancers—those detected between regular screening rounds—can account for up to 21% of breast cancer diagnoses [7]. High false-positive recall rates further compound the issue. A Singaporean study by Ho et al. found that only 7.6% of all mammograms recalled for further evaluation ultimately led to a cancer diagnosis [8]. These unnecessary recalls increase patient anxiety, lead to avoidable procedures, and reduce future screening adherence [9]. Furthermore, diagnostic accuracy varies widely among radiologists, with studies showing detection rates can differ by as much as twofold between general and specialist readers [10].

Artificial intelligence (AI) has emerged as a promising solution to address these challenges. Deep learning models can flag suspicious regions, triage normal exams, and assist in image interpretation, which can potentially improve accuracy and reduce workload. In landmark international studies, AI systems have demonstrated significant reductions in false positives and false negatives. For instance, McKinney et al. showed reductions of up to 9.4% in false negatives and 5.7% in false positives in the U.S. population [11]. Lotter et al. also reported that their AI model outperformed five breast-imaging specialists with a 14% average increase in sensitivity [12]. AI has also shown potential to support single-reader workflows and to safely exclude up to 60% of normal mammograms without compromising sensitivity, thereby improving efficiency [13].

To assess the relevance of these developments in the Singapore context, a recent multi-reader, multi-case study was conducted at the National University Hospital [14]. Seventeen radiologists interpreted 500 de-identified mammograms with and without AI assistance. The study found that while consultant radiologists had comparable accuracy with or without AI (AUROC 0.90), senior residents showed marked improvement when aided by AI, achieving consultant-level performance (AUROC 0.88). Junior residents also demonstrated gains, and the standalone AI system achieved an AUROC of 0.93. Improvements in sensitivity and specificity were most evident among less experienced readers. These findings suggest that AI can help close the experience gap in mammography interpretation and support workforce training and sustainability, which are critical components in a growing screening program. However, the widespread implementation of AI requires substantial investment in software integration, clinical validation, and training. In addition, increased detection of indolent or benign lesions may lead to overdiagnosis and overtreatment [15]. As such, questions remain about the long-term value and cost-effectiveness of AI integration in national screening programs.

To address this gap, we conducted an economic evaluation to evaluate the incremental benefits, harms, and costs associated with AI-assisted mammography in Singapore’s national breast cancer screening program. Our model incorporates real-world screening performance, disease progression, treatment outcomes, and healthcare costs tailored to the Singaporean context. Through this analysis, we aim to provide locally relevant evidence to inform policymaking and guide responsible AI adoption in breast cancer screening.

## 2. Materials and Methods

### 2.1. Population and Setting

This study was reported in accordance with the CHEERS-AI checklist [16], with the completed checklist provided in the Appendix A (Table A1). The target population was Singaporean women aged 50 years, modelled over a 50-year time horizon. This reflects Singapore’s Healthier SG breast cancer screening strategy, which offers biennial mammography to women aged 50–69. A closed cohort design was adopted, whereby a hypothetical population of 10,000 women entered the model at baseline and was followed longitudinally without replenishment. This approach enables the simulation of repeated screening cycles, disease progression, and survival outcomes over time.

### 2.2. Comparator and Intervention

Three screening strategies were compared (Table 1), representing different levels of AI integration:

1. Standard double-reading (comparator): Two radiologists independently interpret each mammogram, with discordant cases resolved by a third reader. This is a non-directive strategy in which full clinical autonomy is retained by human readers;
2. AI-companion: One radiologist is replaced by AI. A radiologist performs the initial read, and discordant cases are arbitrated by another radiologist. Decision-making authority remains with the radiologist, with AI serving as a supportive tool;
3. AI-standalone: The AI system interprets the mammogram without a human reader in the initial decision. This directive strategy leaves no clinical autonomy to the user, with AI directly determining the screening outcome.

**Table 1:** Comparison of AI-standalone and AI-companion reader versus conventional mammogram screening approaches.

Domain	Description
Population	Singaporean women aged 50 to 69 years, consistent with the HealthierSG mammographic screening guidelines. Screening occurs every 2 years.
Intervention	Three breast cancer screening strategies evaluated: (1) AI Standalone (no reading by radiologist), (2) AI as Companion Reader (assisting 1 radiologist). AI systems modelled from published Asian datasets; assumes integration into national workflow.
Comparator	Standard of care: Double reading by two consultant radiologists, with arbitration for discordant cases.
Outcomes	Health: False negatives, false positives, cancers detected, stage distribution shift, False positives requiring unnecessary investigations (including ultrasound and biopsy), Quality-Adjusted Life Years (QALYs) Costs: screening, diagnostics, treatment by stage
Time Horizon & Model	Lifetime horizon: 50 years
Perspective	Health system perspective

All three strategies were applied to the same cohort of women and identical screening interval; only the reading strategy and test performance parameters differed.

The AI intervention evaluated in this study was FxMammo (FathomX), a deep learning-based computer-aided detection and diagnosis (CADe/CADx) system for digital mammography [17]. FxMammo generates lesion probability maps and recall decisions from full-field mammograms and can be implemented either as a companion reader alongside a radiologist or as an automated reader with optional human arbitration. For the purposes of this economic evaluation, diagnostic performance parameters were derived from previously published validation studies of the system and applied as model inputs [14]. The sensitivity and specificity values used were 0.715 and 0.968 for AI-assisted reading and 0.805 and 0.893 for AI-alone interpretation, compared with 0.691 and 0.954 for conventional double reading. Although the algorithm was trained on multinational datasets, Singapore-specific epidemiological, screening, and cost inputs were used to reflect the local population screening context.

### 2.3. Outcomes and Costs

Outcomes included health outcomes—life years (LYs) and quality-adjusted life years (QALYs), and breast cancer deaths averted; diagnostic outcomes—true positives, false positives, and false negatives; and economic outcomes—direct medical costs of screening, diagnosis, treatment, and follow-up. QALYs were calculated by multiplying time spent in each health state by the corresponding utility weight. Stage-specific utility values were adapted from Wong et al. and a Korean cohort. Costs and health outcomes were discounted at 3% annually.

All costs were estimated in 2023 Singapore dollars (SGD) from the healthcare system perspective. Mammography costs were SGD 110 per scan (imaging plus interpretation by two radiologists). For the AI-companion strategy, one radiologist’s fee was replaced by the AI service fee (SGD 5), reducing the per-scan cost to SGD 97.50. For the AI-standalone strategy, radiologist interpretation costs were excluded and replaced with the AI fee, giving SGD 80 per scan. Costs of ultrasound, biopsy, and treatment by stage were obtained from local hospitals and published sources.

### 2.4. Model Structure

**Figure 1:**
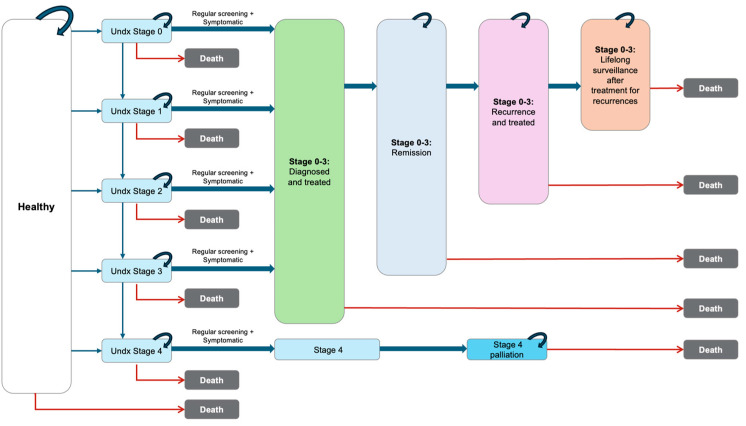
Markov State transition model of breast cancer progression and management. Undx = undiagnosed.

A state-transition Markov model (Figure 1) was developed in R version 4.4.2 to simulate screening outcomes over a 50-year horizon. The model accounted for repeated screening cycles, cancer progression, recurrence, and death from natural causes. Women diagnosed with breast cancer were assumed to receive curative-intent treatment and transition into remission, with recurrences managed in public hospitals under lifelong annual surveillance. Interval cancers were captured as cancers missed at screening or arising between scheduled screens. The model simulated repeated biennial screening cycles and allowed women to transition between health states, reflecting cancer onset and progression, diagnosis, treatment, remission, recurrence, and death from breast cancer or other causes. Women diagnosed with breast cancer were assumed to receive curative-intent treatment and enter a remission state, with any recurrence managed in the public system and followed by lifelong annual surveillance. Interval cancers were modelled as cancers that were either missed at screening or became clinically apparent between scheduled screening rounds. The “undiagnosed” was treated as a time-varying (dynamic) state rather than a fixed subgroup of patients. At any point in time, the undiagnosed state includes individuals with preclinical cancer who have not yet been diagnosed either because their cancer was missed at screening (false negative), because they are in a non-screening year, or because they did not attend screening (non-compliance, assumed 60%). Individuals can leave the undiagnosed state in subsequent cycles through detection at a later screening round, symptomatic presentation as an interval cancer, or incidental diagnosis through other clinical encounters.

### 2.5. Information Source

All unit costs and resource quantities were estimated in Singapore dollars based on 2023 price levels, using data from local public hospitals and the published literature. No currency conversion was required. Parameters were sourced from studies conducted in Asia and from public healthcare institutes in Singapore (Table A2) [18,19,20,21,22,23,24,25,26,27,28,29,30]. All breast cancer related mortality rates were obtained from the Singapore Cancer Registry Annual Report 2015 [20]. Baseline breast cancer prevalence was generated from one year of simulation at age 49. The compliance rate for national screening was assumed to be 40%, rounded from the 37.6% reported in 2022 [31]. To simulate possible changes in compliance, additional scenarios were modelled at 20% and 80%.

Direct medical costs for breast cancer stages 0–IV were obtained from Wong [23], while remission costs were estimated as a percentage of stage-specific direct medical costs. Costs for recurrence treatment were assumed to be the same across stages [30]. The costs of mammography, ultrasound, and biopsies were adapted from local public hospitals. Health utilities were derived from Wong [23], which were adapted from a Korean study [32]. A 3% discount rate was applied to both costs and health outcomes [33].

The cost per mammogram was derived from the baseline cost of SGD 110, which included image acquisition and interpretation by two radiologists. For the single-reader plus AI workflow, the second radiologist’s fee was replaced with the AI service cost of SGD 5, resulting in a per-scan cost of SGD 97.50. For the AI-standalone workflow, radiologist interpretation costs were excluded and replaced by the AI service fee, yielding a total cost of SGD 80 per scan. To ensure face validity, the cost estimates for the three screening strategies were reviewed by six consultant radiologists at the National University Hospital who had participated in the AI related multi-reader studies. These radiologists agreed that the assumptions were reasonable and representative of contemporary clinical practice. The willingness-to-pay (WTP) threshold was set at one gross domestic product (GDP) per capita of approximately SGD 50,000 per QALY gained, in line with thresholds commonly applied in Singapore.

### 2.6. Uncertainty Analysis

Deterministic sensitivity analysis (DSA) varied key parameters by ±10% [34]. Probabilistic sensitivity analysis (PSA) used Gamma distributions for cost parameters and Beta distributions for utilities, with standard deviations set at ±0.05. A total of 10,000 Monte Carlo simulations were performed [35].

## 3. Results

Three breast cancer screening strategies were evaluated: conventional mammography with radiologist double reading, AI-assisted mammography with radiologist arbitration (AI + radiologist), and AI-standalone interpretation with radiologist arbitration. Compared with conventional screening, the AI-assisted strategy demonstrated improved diagnostic performance, with a reduction in false negatives from 144 to 131 and false positives from 1761 to 1225. The number of undiagnosed cases decreased marginally from 1201 to 1191. This strategy also resulted in a shift toward earlier detection, with an increase in early-stage diagnoses from 227 to 231 and a modest change in the early-to-late stage ratio (1.96 vs. 1.95) (Table 2). Compared with conventional double reading, the AI-standalone strategy yielded more true-positive detections (372 vs. 343) and substantially fewer false negatives (85 vs. 144), consistent with higher sensitivity. However, this was accompanied by a marked increase in false positives (4097 vs. 1761), reflecting reduced specificity and a higher recall burden. The total number of undiagnosed cases decreased from 1201 under conventional screening to 1155 with AI-standalone.

From an economic perspective, the AI + Radiologist strategy was the most cost-effective. It provided an additional 15.9 quality-adjusted life years (QALYs) at a total cost of SGD 316,089.60 in savings, resulting in an incremental cost-effectiveness ratio (ICER) of SGD 19,846.08 cost savings per QALY gained. The AI Alone strategy produced the largest health gains, resulting in the least false negative cases, 85. It yielded 218,532.4 QALYs and the greatest reduction in late-stage cancers. However, this was accompanied by a higher total cost of SGD 1.35 million difference, with an ICER of SGD 18,743.39 per QALY gained relative to conventional screening.

**Figure 2:**
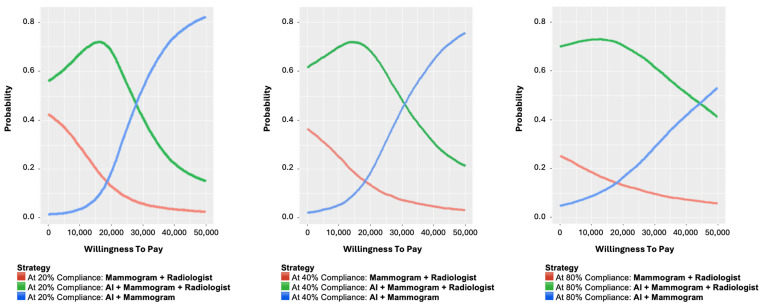
Cost-effectiveness acceptability curves (CEACs) for three breast cancer screening strategies: Conventional mammography with double reading, AI-assisted mammography with radiologist arbitration, and AI-alone interpretation, at varying levels of population compliance (20%, 40%, and 80%).

Across screening compliance levels of 20%, 40%, and 80%, the AI + Radiologist strategy showed the highest probability of being cost-effective at WTP values below approximately SGD 30,000. The probability of cost-effectiveness for this strategy peaked between SGD 10,000 and SGD 25,000, after which it declined as the WTP increased (Figure 2). The AI Alone strategy had a lower probability of cost-effectiveness at lower WTP thresholds but showed a gradual increase as WTP values rose. At WTP thresholds above approximately SGD 35,000 to SGD 40,000, the probability of AI Alone being cost-effective surpassed that of the AI + Radiologist strategy. Conventional mammography consistently exhibited the lowest probability of cost-effectiveness across all WTP thresholds and compliance levels. Its probability declined steadily as WTP increased. Higher participation rates were associated with an increase in absolute false positive numbers but did not materially alter the relative cost-effectiveness ranking of strategies, with the AI-assisted strategy remaining the most cost-effective across all compliance scenarios.

**Figure 3:**
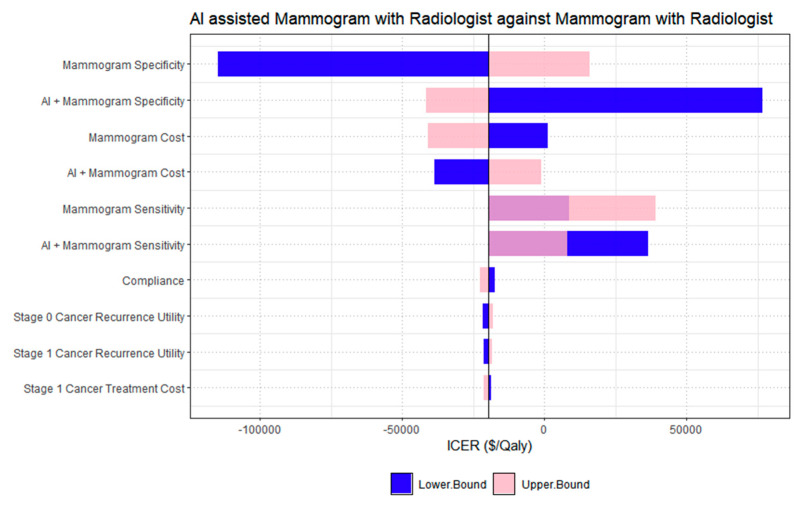
One-way deterministic sensitivity analysis for AI assisted mammogram versus conventional mammography double reading.

**Figure 4:**
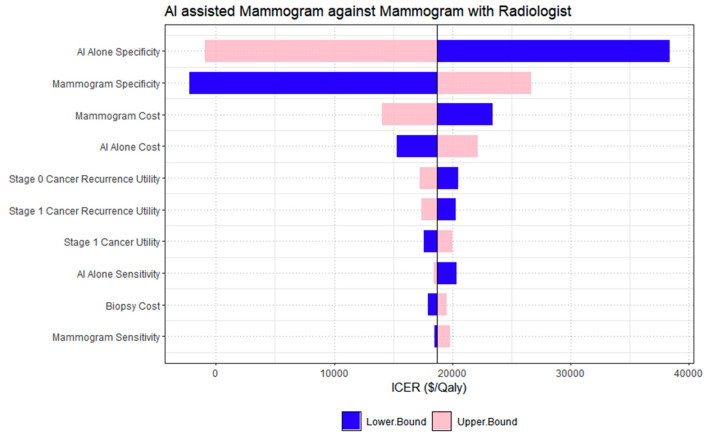
One-way deterministic sensitivity analysis for AI standalone versus conventional mammography double reading.

The one-way deterministic sensitivity analysis in Figure 3 compared the cost-effectiveness of AI-assisted mammography with radiologist arbitration against conventional double-reading mammography. The parameter with the greatest impact on ICER was the specificity of the AI-assisted strategy, followed closely by the specificity of conventional mammography. Variations in these parameters produced the widest ICER ranges, spanning from dominant (cost-saving) to cost-increasing outcomes, indicating that diagnostic specificity is a key driver of cost-effectiveness due to its influence on false-positive rates and subsequent downstream costs. Screening cost parameters, including the costs of both conventional and AI-assisted mammography, also contributed meaningfully to ICER variability, though to a lesser extent than specificity. Parameters related to sensitivity and compliance showed moderate influence on ICER estimates, while those associated with cancer recurrence utility values and stage 1 treatment costs had comparatively limited impact. In the comparison between AI-alone mammography and conventional mammography interpreted by radiologists (Figure 4), the one-way deterministic sensitivity analysis identified AI-alone specificity as the most influential parameter affecting the ICER. Variations in this parameter resulted in the broadest range of ICER values, highlighting its strong influence on model outcomes. Specificity of conventional mammography also had a substantial impact, reinforcing the importance of diagnostic accuracy, particularly in reducing false positives, in determining cost-effectiveness. Parameters related to screening costs, including the cost of mammography and the AI-alone intervention, also affected the ICER, though to a lesser extent than specificity. Other parameters, such as utilities for cancer recurrence and stage 1 cancer, AI-alone sensitivity, biopsy cost, and mammogram sensitivity, also contributed to ICER variation but had comparatively smaller effects.

## 4. Discussion

The integration of artificial intelligence into breast cancer screening holds significant promise for enhancing cancer detection while preserving low false-positive rates. Evidence from the recent MASAI trial (screening performance and characteristics of breast cancer detected in the Mammography Screening with Artificial Intelligence trial) demonstrated that AI-supported screening significantly increased cancer detection rates without compromising specificity, while reducing radiologist workload by 44% [36]. However, moving from proof-of-concept to routine clinical adoption requires not only clinical validation but also rigorous economic evaluation to determine whether these technologies provide sufficient value within the constraints of existing healthcare systems. This study showed that AI-augmented breast cancer screening strategies improved outcomes compared with conventional double reading. The AI-alone strategy achieved the greatest health gains, yielding 218,532.4 QALYs and the lowest false-negative count (85 versus 144), reflecting its higher sensitivity. However, these benefits were offset by a substantially higher false-positive burden (4097) and increased total costs (+SGD 1.35 million), resulting in an ICER of SGD 18,743.39 per QALY and raising clinical and system-level concerns related to downstream investigations and resource utilisation. In contrast, the AI-assisted strategy demonstrated the most favourable economic profile, delivering 15.9 additional QALYs while remaining cost-saving SGD −19,846.08 per QALY and reducing false positives to 1225. Sensitivity analyses identified diagnostic specificity, screening costs, and early-stage detection as key drivers of economic value.

To our knowledge, this is one of the first studies in Asia to conduct a comprehensive cost-effectiveness evaluation of AI-enhanced breast cancer screening under real-world conditions. By integrating both diagnostic performance and economic outcomes, this study provided robust, locally relevant evidence that addresses the critical question of whether AI deployment in screening is sustainable and beneficial at a population level. Our findings suggest that incorporating AI into breast cancer screening workflows can improve population health outcomes compared to conventional screening, primarily through gains in quality-adjusted life years driven by earlier cancer detection, reduced morbidity, and improved quality of life. Importantly, our modelling demonstrates that both AI-assisted and AI-standalone strategies are cost-effective alternatives to conventional double-reading. These results not only support the adoption of AI in screening but also highlight the flexibility of context-sensitive implementation, allowing health systems to adapt the level of automation based on local resources, regulatory requirements, and clinical infrastructure [37,38]. By explicitly linking diagnostic gains with economic sustainability, this study provides critical evidence to guide policy, reimbursement, and resource allocation decisions in the region.

Among the evaluated strategies, the AI-alone model produced the largest health gains, with an estimated 72 additional QALYs per 10,000 women screened, driven by its higher sensitivity (0.805). However, this came at the expense of lower specificity (0.893), more false positives, and higher overall costs. In contrast, the AI-assisted model (AI + radiologist) achieved a more favourable balance, with 15.9 additional QALYs and cost savings of SGD 19,846 per QALY compared to conventional double-reading. This made it the most cost-effective option across low to intermediate willingness-to-pay thresholds. The AI-standalone strategy was associated with an approximately threefold increase in false-positive recalls compared with conventional screening. This substantial increase has direct clinical and ethical implications, given the well-documented cascading consequences of false positives, including additional diagnostic imaging, invasive biopsies, psychological distress, patient anxiety, and increased healthcare resource utilisation. In this context, the elevated false-positive burden represents not only an economic cost but also a key practical and ethical barrier to real-world adoption of fully automated screening strategies. On the other hand, AI-assisted screening represents a more economically viable and pragmatic transitional model for adoption in breast cancer screening programs.

At higher WTP thresholds (>SGD 40,000), fully automated strategies may become attractive, although further evidence such as interval cancer rates and long-term safety will be needed to support widespread implementation. The preference for AI-assisted strategies is consistent with international evidence showing that hybrid human–AI models achieve a favourable balance of diagnostic accuracy, cost-effectiveness, and clinical acceptability. For example, the MASAI trial reported that AI-supported triage improved cancer detection and reduced radiologist workload by 44% without compromising safety [39]. Similarly, Armando et al. found that using AI as a second reader was a cost-effective strategy for the UK National Health Service, provided that diagnostic non-inferiority was maintained [40]. Collectively, these results support a phased and context-sensitive approach to AI adoption in breast cancer screening.

Sensitivity analyses identified test specificity as the dominant driver of cost-effectiveness across AI-based breast cancer screening strategies, consistent with prior studies demonstrating the disproportionate downstream costs associated with false positives, including additional imaging, biopsies, psychological distress, and increased healthcare utilization [41]. This effect was particularly evident in AI-alone models, where small reductions in specificity substantially increased recall rates and resource burden, a pattern also observed in other screening programmes such as diabetic retinopathy [42]. Across screening compliance levels (20%, 40%, and 80%), the AI-assisted strategy remained the most cost-effective at lower to moderate willingness-to-pay (WTP) thresholds (<SGD 30,000 per QALY), reflecting its favourable balance between costs and outcomes. At higher WTP thresholds (>SGD 35,000–40,000), the AI-alone strategy became more economically attractive due to greater cancer detection and QALY gains, although real-world adoption remains constrained by ethical, legal, and trust considerations related to fully automated decision-making. Survey findings similarly indicate a preference for AI-assisted rather than fully autonomous screening, with accountability expected to remain with healthcare providers [38,43]. Collectively, these findings support hybrid AI-assisted models as the most acceptable and implementable pathway for population-based breast screening.

Cross-study comparisons reinforce that the economic value of AI in breast cancer screening depends primarily on how AI is integrated into clinical workflows rather than algorithm performance alone. In the Swedish AI-DM evaluation, AI functioned as a triage tool that selectively replaced the second radiologist in low-risk examinations while retaining double reading for higher-risk cases [44]. This selective labour substitution resulted in 10.8 additional QALYs per 1000 women screened and a net cost saving of EUR 59,320 (≈SGD 86,000), rendering the AI-assisted strategy cost-saving compared with conventional double reading In contrast, our AI-assisted strategy assumed systematic replacement of the second reader across all examinations without risk-based triage. Despite these operational differences, both studies demonstrated cost savings and QALY gains relative to conventional double reading, driven by reduced radiologist manpower requirements without substantial loss of diagnostic specificity. Conversely, models involving greater technological intensification, such as the U.S. AI-assisted digital breast tomosynthesis (DBT) cohort and our AI-standalone strategy, showed that increased automation does not necessarily translate into economic efficiency. In the DBT model, AI-assisted screening generated only modest health gains (3.09 additional QALYs per 1000 women) but incurred substantially higher lifetime costs (USD 936,430 per 1000 women; ≈SGD 1.26 million), resulting in an ICER of approximately USD 303,279 per QALY gained (≈SGD 409,000 per QALY), exceeding conventional willingness-to-pay thresholds [45]. Although these approaches produced greater cancer detection and incremental QALYs, they were associated with higher costs due to false positives, overdiagnosis, and downstream treatment expenditure, resulting in less favourable cost-effectiveness profiles. Collectively, findings across Singaporean, European, and U.S. studies suggest a consistent policy signal: AI provides the greatest economic value when used to augment or partially substitute human readers within hybrid screening pathways rather than as fully autonomous systems or adjuncts to already costly imaging technologies. This supports phased implementation strategies prioritising human-in-the-loop AI-assisted mammography as the most scalable and economically sustainable approach for population screening.

While this model-based economic evaluation provides important insights, several limitations should be considered. The analysis was restricted to women aged 50–69 years in accordance with Singapore’s national screening programme and therefore does not capture outcomes in younger or higher-risk populations where AI performance remains less well validated. Fixed sensitivity and specificity values were assumed for the FxMammo algorithm, which may not fully reflect real-world variability due to software updates, imaging protocols, or population differences; ongoing post-deployment monitoring and adaptive recalibration would be required in practice. Mortality inputs were derived from the Singapore Cancer Registry Annual Report 2015, which provides the most detailed age-specific estimates required for model parameterisation, as more recent reports present aggregated statistics without sufficient granularity. Although treatments have evolved, much of the mortality benefit of screening arises from earlier stage detection, and survival outcomes in Singapore are already favourable; therefore, temporal improvements in therapy are unlikely to materially alter comparative differences between screening strategies. Residual uncertainty from historical mortality inputs was explored in sensitivity analyses, with conclusions remaining robust. The model also does not explicitly quantify the psychological harms associated with false negatives or overdiagnosis, including patient anxiety and distress, although downstream healthcare costs related to additional investigations, recalls, biopsies, and treatment were incorporated within the economic analysis. The AI-alone strategy improved sensitivity but reduced specificity, whereas AI-assisted reading preserved specificity through radiologist arbitration, highlighting the need for careful threshold optimisation using local audit data. Population heterogeneity was not explicitly modelled, including differences by ethnicity, socioeconomic status, or comorbidity, which may influence both access and algorithm performance, and the potential for algorithmic drift over time requires ongoing monitoring to ensure equity. Assumptions of full screening adherence and timely follow-up may overestimate effectiveness, particularly given variations in uptake and the presence of opportunistic screening outside the national programme. Operational factors such as PACS integration, workflow redesign, training requirements, and regulatory approval were not explicitly incorporated but remain critical for implementation. The AI-standalone strategy further assumes diagnostic autonomy without modelling medico-legal or patient acceptance barriers that may affect real-world adoption. Despite these limitations, the study provides a robust foundation for future implementation research, underscoring the need for ongoing evaluation, local validation, and responsible integration of AI within established screening programmes.

## 5. Conclusions

AI integration into breast cancer screening has the potential to improve health outcomes and system efficiency compared with conventional double reading. Hybrid human-in-the-loop implementation, in which AI supports radiologist decision-making, appears to provide the most favourable balance between clinical benefit, cost-effectiveness, and operational feasibility without compromising diagnostic performance. In contrast, fully automated AI approaches may offer greater detection gains but are associated with higher costs and downstream resource implications, warranting further optimisation and evaluation before widespread adoption. Overall, these findings support a phased implementation strategy prioritising AI-assisted workflows as the most practical pathway for near-term population screening programmes.

## Figures and Tables

**Table 2:** Cumulative diagnostic outcomes, costs, and health effects for a cohort of 10,000 women over a 50-year time horizon (40% screening compliance).

Metric	Conventional Strategy: 2 Radiologists	Hybrid Strategy: 1 Radiologist & AI	Standalone Strategy: AI Alone
Total Mammograms Completed	38,628	38,621	38,598
Total True Positive mammograms	343	349	372
Total True Negative mammograms	36,379	36,915	34,044
Total False Positive mammograms	1761	1225	4096
Total False Negative mammograms	144	131	85
Total Undiagnosed Cancer Cases	1201	1191	1155
Early Stage Cancer Patients	227	231	246
Late Stage Cancer Patients	116	118	126
Total Cost	19,179,907.50	18,863,817.90	20,529,718.70
Total QALYS (SGD)	218,460.4	218,476.3	218,532.4
ICER	-	−19,846.08	18,743.39

Note: Values represent cumulative diagnostic events accrued over the 50-year time horizon. Because individuals may undergo multiple screening cycles and diagnostic assessments, categories including true positives, true negatives, false positives, and false negatives are not mutually exclusive and therefore do not sum to the initial cohort size of 10,000 women. Each screening attendance generates a diagnostic classification, meaning that a single individual may contribute multiple outcomes across time. Consequently, counts reflect cumulative screening events rather than unique individuals.

## Data Availability

All data generated or analysed during this study are included in this published article.

## Appendix A

**Table A1:** CHEERS checklist.

Section/Topic	No.	Guidance for Reporting	AI Elaboration	Reported in Section
Title	1	Identify the study as an economic evaluation and specify the interventions being compared.	Indicate that the intervention involves an AI component that is under evaluation.	1
Abstract	2	Provide a structured summary that highlights context, key methods, results, and alternative analysis.	Specify the purpose of the intervention with an AI component, and the AI technique used.	1
Background and study objectives	3	Give the context for the study, the study question, and its practical relevance for decision making in policy or practice.		2
Health economic analysis plan	4	Indicate whether a health economic analysis plan was developed and where available.		3
Study population	5	Describe characteristics of the study population (such as age range, demographics, socioeconomic, or clinical characteristics).		3
Setting and location	6	Provide relevant contextual information that may influence findings.		3
Comparators	7	Describe the interventions or strategies being compared and why chosen.	Describe key details of the AI component of the intervention (and comparators, if appropriate), including: (a) the classification by intended purpose and risk tier (for digital health technologies); (b) the AI technique used; (c) whether it is “locked” (static) or adaptive; (d) the version under evaluation; (e) the purpose of the intervention, including its potential impact on care; (f) the intended user(s), and how users interact with it; (g) additional requirements to use it; (h) how it is expected to provide benefit over the standard of care.	3
User autonomy	AI 1	Indicate whether the AI intervention (and comparators, if appropriate) is directive, or whether the user(s) retains autonomy to make the care decision.		3
Perspective	8	State the perspective(s) adopted by the study and why chosen.		4
Time horizon	9	State the time horizon for the study and why appropriate.		4
Discount rate	10	Report the discount rate(s) and reason chosen.		4
Selection of outcomes	11	Describe what outcomes were used as the measure(s) of benefit(s) and harm(s).	Describe whether the measure(s) chosen to indicate the benefits and harms of the AI intervention (and comparators) relates to health outcomes, diagnostic outcomes, process outcomes, or other/multiple outcomes.	4
Measurement of outcomes	12	Describe how outcomes used to capture benefit(s) and harm(s) were measured.	For model-based analysis, describe any assumptions used to inform the potential benefit(s) and harm(s) of the AI intervention in the model (and comparators, if appropriate). Describe the plausibility of analyst assumptions, citing any supportive evidence.	4
Measurement of AI effect	AI 2	Describe the data sources (assessment studies) for the AI intervention’s impact on outcomes.		2
Measurement of AI learning over time	AI 3	If the AI intervention (and comparators, if appropriate) learns over time, explain how this affects its performance at the individual level and how this was measured.		N.A.
Development of AI component	AI 4	Describe how the AI component of the intervention (and comparators, if appropriate) was developed, including the training data used and how errors and biases were identified, or cite a source that provides this information.		3
Validation of AI component	AI 5	Describe how the AI component of the intervention (and comparators, as appropriate) and its performance estimates were validated, or cite a source that provides this information.		2,3
Health benefit	AI 6	Describe how the AI intervention (and comparators, if appropriate) could directly or indirectly provide a health benefit.		3
Population differences	AI 7	Describe important differences between the data sources (assessment studies) for the AI intervention’s impact on outcomes and the data set that was used to develop the AI intervention (training data set).		3
Valuation of outcomes	13	Describe the population and methods used to measure and value outcomes.		3
Measurement and valuation of resources and costs	14	Describe how costs were valued. Describe the purchase cost of the AI intervention (and comparators, if appropriate) and what it is composed of. Describe any additional implementation and maintenance costs.		3
Currency, price date, and conversion	15	Report the dates of the estimated resource quantities and unit costs, plus the currency and year of conversion.		3
Rationale and description of model	16	If modeling is used, describe in detail and why used. Report if the model is publicly available and where it can be accessed. Describe if the AI component of the intervention has influenced the choice of health economic model and explain why.		3
Analytics and assumptions	17	Describe any methods for analysing or statistically transforming data, any extrapolation methods, and approaches for validating any model used.		3
Modelling of AI learning over time	AI 8	If the AI intervention (and comparators, if appropriate) learns over time at the individual level, describe any assumptions used to model how this learning affects its performance over time.		NA
Characterizing heterogeneity	18	Describe any methods used for estimating how the results of the study vary for subgroups.		3
Characterizing distributional effects	19	Describe how impacts are distributed across different individuals or adjustments made to reflect priority populations.		3
Characterizing uncertainty	20	Describe methods to characterize any sources of uncertainty in the analysis.		3
Approach to engagement	21	Describe any approaches to engage patients or service recipients, the general public, communities, or stakeholders (such as clinicians or payers) in the design of the study.		1
Study parameters	22	Report all analytic inputs (such as values, ranges, and references) including uncertainty or distributional assumptions.		3
Summary of main results	23	Report the mean values for the main categories of costs and outcomes of interest and summarize them in the most appropriate overall measure.		7
Effect of uncertainty	24	Describe how uncertainty about analytic judgments, inputs, or projections affect findings. Report the effect of choice of discount rate and time horizon, if applicable.		7
Impact of AI uncertainty	AI 9	Indicate the extent to which features of the AI intervention may contribute to increased uncertainty about its cost-effectiveness.		7
Effect of engagement	25	Report on any difference patient/service recipient, general public, community, or stakeholder involvement made to the approach or findings of the study.		7
Study findings, limitations, generalizability, and current knowledge	26	Report key findings, limitations, ethical or equity considerations not captured, and how these could affect patients, policy, or practice.	Comment on potential biases associated with the AI intervention (e.g., algorithmic bias) and implications for the generalizability and interpretation of results (e.g., reinforcing existing health inequalities).	11
Implementation of AI	AI 10	Comment on any requirements needed to integrate the AI intervention (and comparators, as appropriate) into practice, and other implementation considerations relating to the AI component of the intervention, including implications for the interpretation of cost-effectiveness results.		11
Source of funding	27	Describe how the study was funded and any role of the funder in the identification, design, conduct, and reporting of the analysis.		12
Conflicts of interest	28	Report authors conflicts of interest according to journal or International Committee of Medical Journal Editors requirements.		12

## Appendix B

**Table A2:** Input parameters for the cost-effectiveness model.

Variables	Baseline	Minimum Within SD	Maximum Within SD	Distribution	Reference Link
**Age Specific Incidence Rates**					https://www.restoredcdc.org/www.cdc.gov/united-states-cancer-statistics/publications/metastatic-breast-cancer.html (accessed on 28 February 2026)
50–54	0.004983	0.00473385	0.00523215	Beta	
55–59	0.006058	0.0057551	0.0063609	Beta	
60–64	0.007765	0.00737675	0.00815325	Beta	
65–100	0.009771	0.00928245	0.01025955	Beta	
**Age Specific Death Rates**					https://www.singstat.gov.sg/-/media/files/publications/population/excel/lifetable2003-2024.ashx (accessed on 28 February 2026)
50–54	0.0016	0.00152	0.00168	Beta	
55–59	0.0024	0.00228	0.00252	Beta	
60–64	0.0039	0.003705	0.004095	Beta	
65–69	0.0066	0.00627	0.00693	Beta	
70–74	0.0109	0.010355	0.011445	Beta	
75–79	0.0188	0.01786	0.01974	Beta	
80–84	0.0389	0.036955	0.040845	Beta	
85–89	0.0743	0.070585	0.078015	Beta	
90–100	0.1528	0.14516	0.16044	Beta	
**Stage Specific Mortality Rates**					https://www.nrdo.gov.sg/docs/librariesprovider3/Publications-Cancer/cancer-registry-annual-report-2015_web.pdf?sfvrsn=10 (accessed on 28 February 2026)
Stage 0	0.00404	0.003838	0.004242	Beta	
Stage 1	0.02	0.019	0.021	Beta	
Stage 2	0.044	0.0418	0.0462	Beta	
Stage 3	0.083	0.07885	0.08715	Beta	
Stage 4	0.268	0.2546	0.2814	Beta	
**Stage specific check-up Rates**					https://www.nrdo.gov.sg/docs/librariesprovider3/Publications-Cancer/cancer-registry-annual-report-2015_web.pdf?sfvrsn=10 (accessed on 28 February 2026)
Stage 0	0.004	0.0038	0.0042	Beta	
Stage 1	0.004	0.0038	0.0042	Beta	
Stage 2	0.014	0.0133	0.0147	Beta	
Stage 3	0.38	0.361	0.399	Beta	
Stage 4	1	0.95	1	Beta	
**Stage Specific Recurrence Rates**					
Stage 0	0.002	0.0019	0.0021	Beta	https://pubmed.ncbi.nlm.nih.gov/34406870/ (accessed on 28 February 2026)
Stage 1	0.0236	0.02242	0.02478	Beta	https://pubmed.ncbi.nlm.nih.gov/20607258/ (accessed on 28 February 2026)
Stage 2	0.0763	0.072485	0.080115	Beta	
Stage 3	0.1453	0.138035	0.152565	Beta	
**Stage Specific Utility Values**					https://scholarbank.nus.edu.sg/handle/10635/166355 (accessed on 28 February 2026)
Stage 0	0.731	0.69445	0.76755	Beta	
Stage 1	0.731	0.69445	0.76755	Beta	
Stage 2	0.731	0.69445	0.76755	Beta	
Stage 3	0.599	0.56905	0.62895	Beta	
Stage 4	0.352	0.3344	0.3696	Beta	
**Stage Specific Escalation Rates**					https://www.nrdo.gov.sg/docs/librariesprovider3/Publications-Cancer/cancer-registry-annual-report-2015_web.pdf?sfvrsn=10 (accessed on 28 February 2026)
Stage 0 to Stage 1	0.1	0.095	0.105	Beta	
Stage 1 to Stage 2	0.06	0.057	0.063	Beta	
Stage 2 to Stage 3	0.11	0.1045	0.1155	Beta	
Stage 3 to Stage 4	0.15	0.1425	0.1575	Beta	
**Stage Specific Recurrence Utility Values**					https://www.oncotarget.com/article/16985/text/ (accessed on 28 February 2026)
Stage 0	0.73	0.6935	0.7665	Beta	
Stage 1	0.73	0.6935	0.7665	Beta	
Stage 2	0.73	0.6935	0.7665	Beta	
Stage 3	0.58	0.551	0.609	Beta	
**Long Term Care Specific Utility Values**					https://ascopubs.org/doi/10.1200/JCO.2006.10.4190 (accessed on 28 February 2026)
Stage 0	0.79	0.7505	0.8295	Beta	
Stage 1	0.79	0.7505	0.8295	Beta	
Stage 2	0.79	0.7505	0.8295	Beta	
Stage 3	0.79	0.7505	0.8295	Beta	
Stage 4	0.352	0.3344	0.3696	Beta	
**Stage Specific Direct Medical Cost**					https://scholarbank.nus.edu.sg/handle/10635/166355 (accessed on 28 February 2026)
Stage 0	19,759.37698	17,783.43928	21,735.31468	Gamma	
Stage 1	37,970.27732	34,173.24958	41,767.30505	Gamma	
Stage 2	53,567.78373	48,211.00536	58,924.56211	Gamma	
Stage 3	68,168.28852	61,351.45967	74,985.11738	Gamma	
Stage 4	74,887.86218	67,399.07596	82,376.64839	Gamma	
**Stage Specific Remission Cost**					https://pmc.ncbi.nlm.nih.gov/articles/PMC4822976/ (accessed on 28 February 2026)
Stage 0	881.3300622	793.197056	969.4630684	Gamma	
Stage 1	1712.06321	1540.856889	1883.269531	Gamma	
Stage 2	2415.347957	2173.813162	2656.882753	Gamma	
Stage 3	3696.525508	3326.872957	4066.178059	Gamma	
Stage 4	11,383.47	10,245.123	12,521.817	Gamma	
Recurrence	13,240.15	11,916.135	14,564.165	Gamma	
**Screening Metrics**					https://papers.ssrn.com/sol3/papers.cfm?abstract_id=4664541 (accessed on 28 February 2026)
**Sensitivity**					
Mammogram + Radiologist	0.691	0.6219	0.7601	Beta	
AI + Mammogram + Radiologist	0.715	0.6435	0.7865	Beta	
AI + Radiologist	0.805	0.7245	0.8855	Beta	
**Specificity**					
Mammogram + Radiologist	0.954	0.8586	0.99	Beta	
AI + Mammogram + Radiologist	0.968	0.8712	0.99	Beta	
AI + Radiologist	0.893	0.8037	0.9823	Beta	
**Screening Cost**					https://papers.ssrn.com/sol3/papers.cfm?abstract_id=4664541 (accessed on 28 February 2026)
Mammogram + Radiologist	110	99	121	Gamma	
AI + Mammogram + Radiologist	97.5	87.75	107.25	Gamma	
AI + Radiologist	80	72	88	Gamma	
Biopsy	1075	967.5	1182.5	Gamma	https://isomer-user-content.by.gov.sg/3/a7305a12-e9c2-4d88-ad13-ed753d054416/MOH-Fee-Benchmarks-(wef-1-Jan-2025)-Publication.xlsx (accessed on 28 February 2026)
Ultrasound	230	207	253	Gamma	https://bmchealthservres.biomedcentral.com/articles/10.1186/s12913-021-06396-2 (accessed on 28 February 2026)
Compliance	0.4	0.36	0.44	Beta	https://ink.library.smu.edu.sg/soss_research/2180/#:~:text=The%20objective%20of%20the%20Singapore,mammogram%20at%20the%20current%20rates (accessed on 28 February 2026).
